# Retention of zirconia crowns to titanium bases with straight versus angled screw access channels: an invitro study

**DOI:** 10.1186/s12903-023-03177-7

**Published:** 2023-07-07

**Authors:** Eman Assem Ibrahim, Mohamed Moataz Khamis, Salah Ezzelarab, Ahmed M. Abdelhamid

**Affiliations:** Department of Prosthodontics, Faculty of Dentistry, Alexandria University, 1st floor, Champollion Street – Azarita, Alexandria, Egypt

**Keywords:** Angled screw access channel abutment, Retention, Dental implants, Self-adhesive resin cement

## Abstract

**Background:**

The aim of this study was to assess the impact of abutments with angled screw access channel on the retention of zirconia crowns.

**Methods:**

Seven implant replicas were inserted in epoxy resin blocks. Fourteen zirconia crowns for central incisor tooth were digitally fabricated and cemented to titanium bases (Ti-bases) with resin cement. Titanium bases were categorized into 2 groups (*n* = 7). Control group (Group STA) included straight screw access channel abutments. Study group (Group ASC) included angled screw access channel abutments. Following aging (5 °C-55 °C, 60 s; 250,000 cycles, 100 N, 1.67 Hz), the pull-off forces (N) were recorded by using retention test (1 mm/min). Types of failure were defined as (Type 1; Adhesive failure when luting agent predominantly remained on the Ti-base surface (> 90%); Type 2; Cohesive failure when luting agent remained on both Ti-base and crown surfaces; and Type 3; Adhesive failure when luting agent predominantly remained on the crown (> 90%). Statistical analysis was conducted by using (IBM SPSS version 28). Normality was checked by using Shapiro Wilk test and Q-Q plots. Independent t-test was then used to analogize the groups.

**Results:**

Mean ± standard deviation of retention force records ranged from 1731.57 (63.68) N (group STA) to 1032.29 (89.82) N (group ASC), and there was a statistically significant variation between the 2 groups (*P* < .05). Failure modes were Type 2 for group STA and Type 3 for group ASC.

**Conclusions:**

The retention of zirconia crowns to abutments with a straight screw access channel is significantly higher than abutments with angled screw access channel.

## Background

The management of dental situations with screw-retained restorations has been applicable with the spread of abutment with Angled Screw access Channel (ASC) [1]. In the esthetic zone and tilted anterior maxilla, it may be required to place the crown at an inclination to the implant long axis. This leads to the placement of screw access channel on the labial side of the restoration [2]. However, systems that have been recently presented, can permit correction of unfavourable angulation.

The screw access channel of the ASC might be located anywhere between 0 and 25° from the implant long axis [3, 4] Ball hexagon driver can engage the specially designed abutment screw of the ASC to apply the recommended torque values [4].

The ASC abutment has larger screw access opening and fewer axial walls than the straight abutment as a reason of the greater angulation. Therefore, the surface area between the abutment and the crown is reduced.

Recently, titanium bases (Ti-bases) have become a popular part in the digital workflow of implant supported restorations. One advantage is that the crown and the Ti-base can be extra-orally bonded together and then that one piece restoration can be screw retained to the implant [5]. Therefore, the remaining cement is restrained, and the risk of biological complications is reduced [6] Ti-bases encompassing ASC are now available [7].

Moreover, restorations with angled screw channel can be digitally designed and fabricated through the advancements of the software programs and implant systems [7].

Retention force is directly related to the abutment’s surface area. The geometry of the abutment may be a priority to chemical bonding as it provides more retention [5]. The retentiveness of zirconia can be significantly improved by increasing height of the abutment as well as the number of axial walls as they provide more surface area [8–10] It has been stated that, retention of zirconia crowns is directly correlated with Ti-base height, Ti-base blasting or silica coating and chemical bonding [11]. The successful outcome of the Ti-base concept is based on the fact that Ti-base and the ceramic superstructure have a stable bonded interface [12].

Therefore, this in vitro study aimed to assess the impact of abutments with angled screw access channel on the retention force applied on zirconia crowns. The null hypothesis was that the angled screw channel Ti-bases have no effect on the retention of adhesively bonded zirconia crowns.

## Methods

### Preparation of sample

Seven implant analogues were embedded into epoxy resin blocks. Implant replicas were maintained in position with a guide pin and connected to a laboratory surveyor. The surveyor was used to permit the fixation of the implant replicas into the resin blocks [3]. Implants were positioned at a distance of 3 mm between the cervical portion of the implant and upper surface of the resin.

**Fig. 1:**
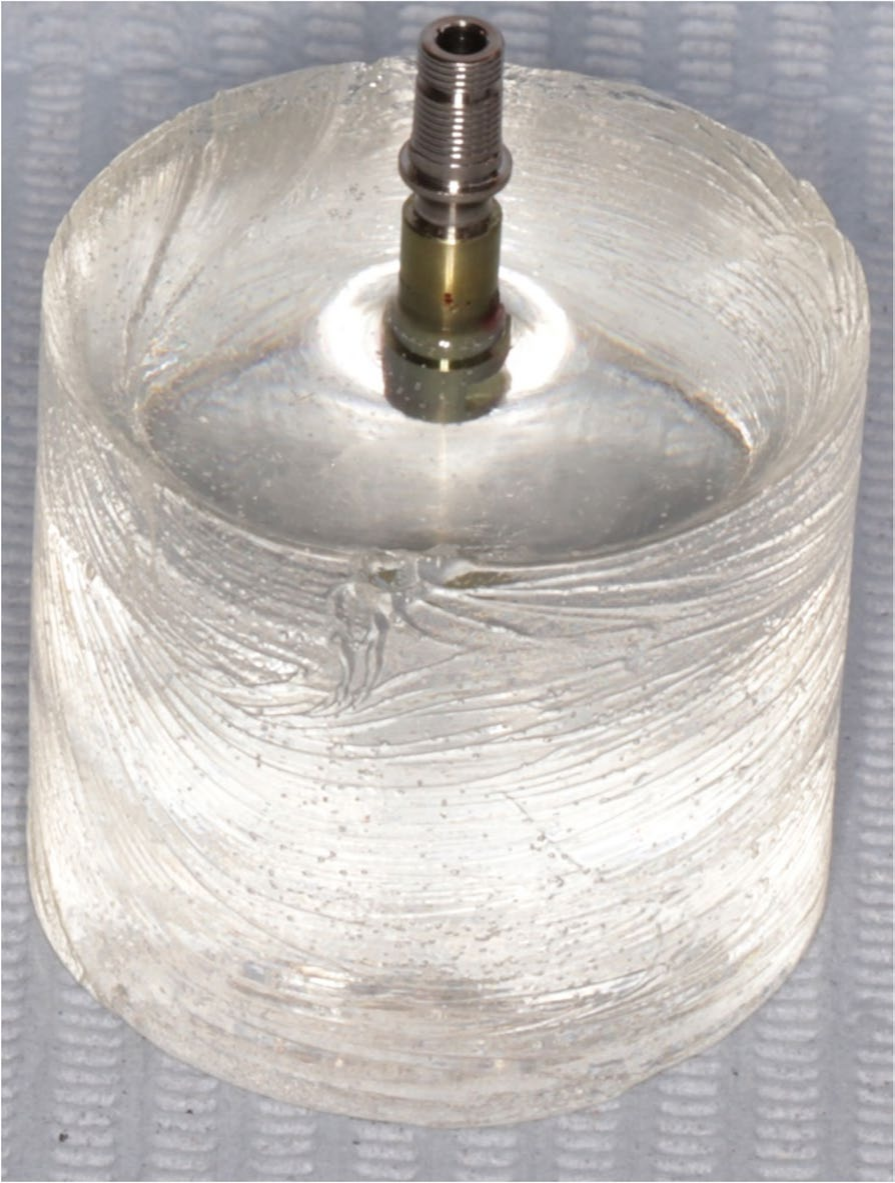
Group STA (control group) included straight screw access channel Ti-bases

**Fig. 2:**
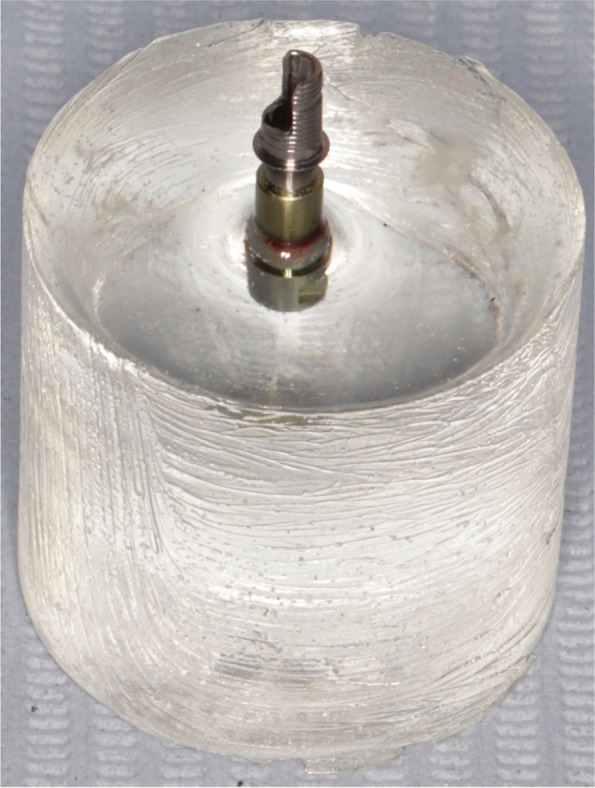
Group ASC (study group) included angled screw access channel Ti-bases

The sample size was calculated presuming 5% alpha error and 80% study power. Sample size was based on Rosner’s method [13] calculated by G* power 3.1 [14]. The total sample size was determined as (*n* = 14) samples that were grouped into 2 groups based on the angulation of the screw-access channel. Group STA (control group) included (*n* = 7) straight screw access channel Ti bases (Fig. 1). Group ASC (study group) included (*n* = 7) angled screw access channel Ti bases [15] (Fig. 2).

**Fig. 3:**
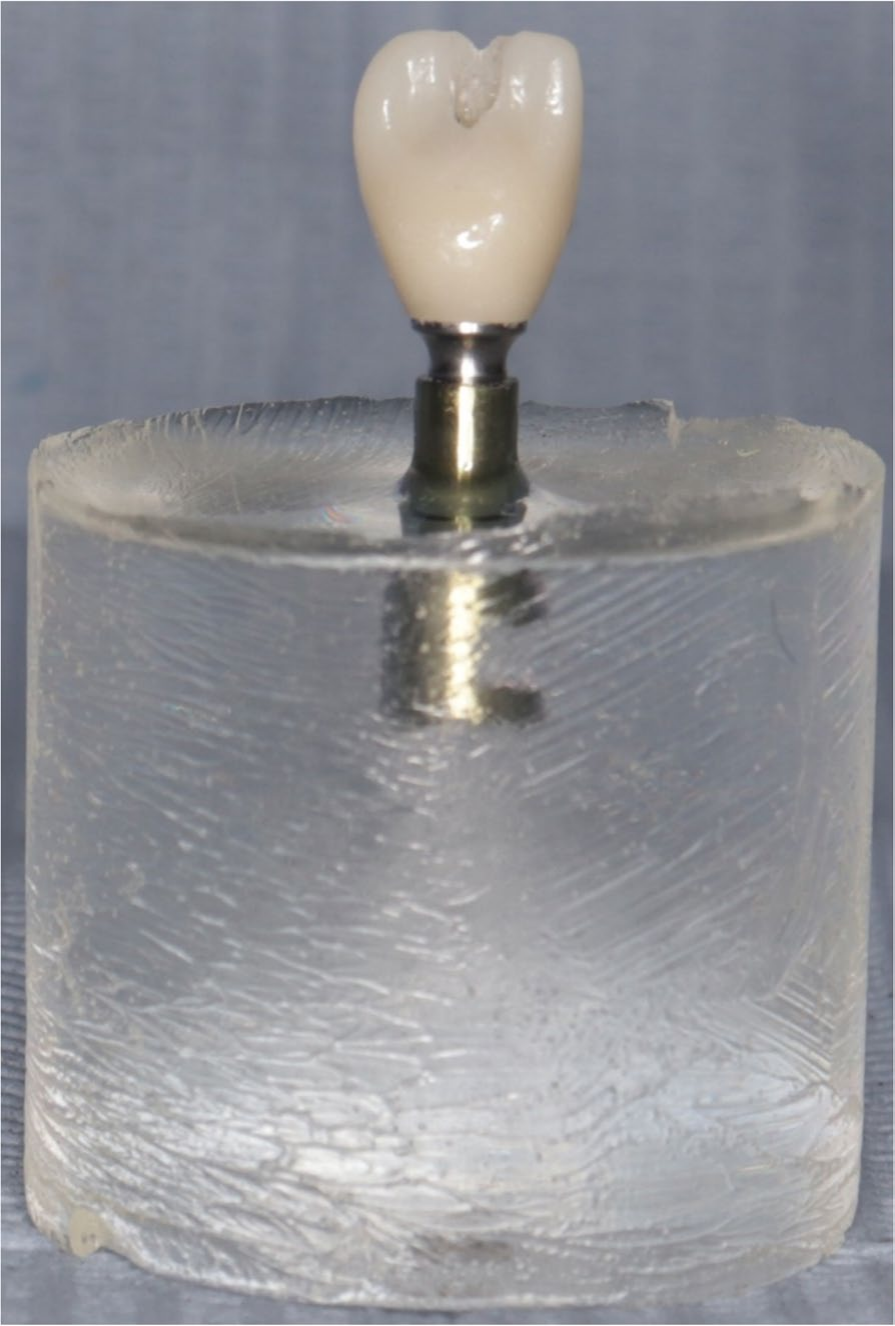
Screw access hole of restoration for group STA was located on incisal edge

Single zirconia crown for central incisor teeth was digitized for each sample. For Group STA, straight screw access channel Ti-base (GM exact titanium base with removable screw, Neodent) of size 4.5 × 6 × 2.5 mm was selected from the library on (Ceramill, Amann Girrbach) CAD software. The screw access hole of the straight Ti-base is located at the incisal edge. The screw access hole of the restoration was designed to be located near the incisal edge (Fig. 3).

**Fig. 4:**
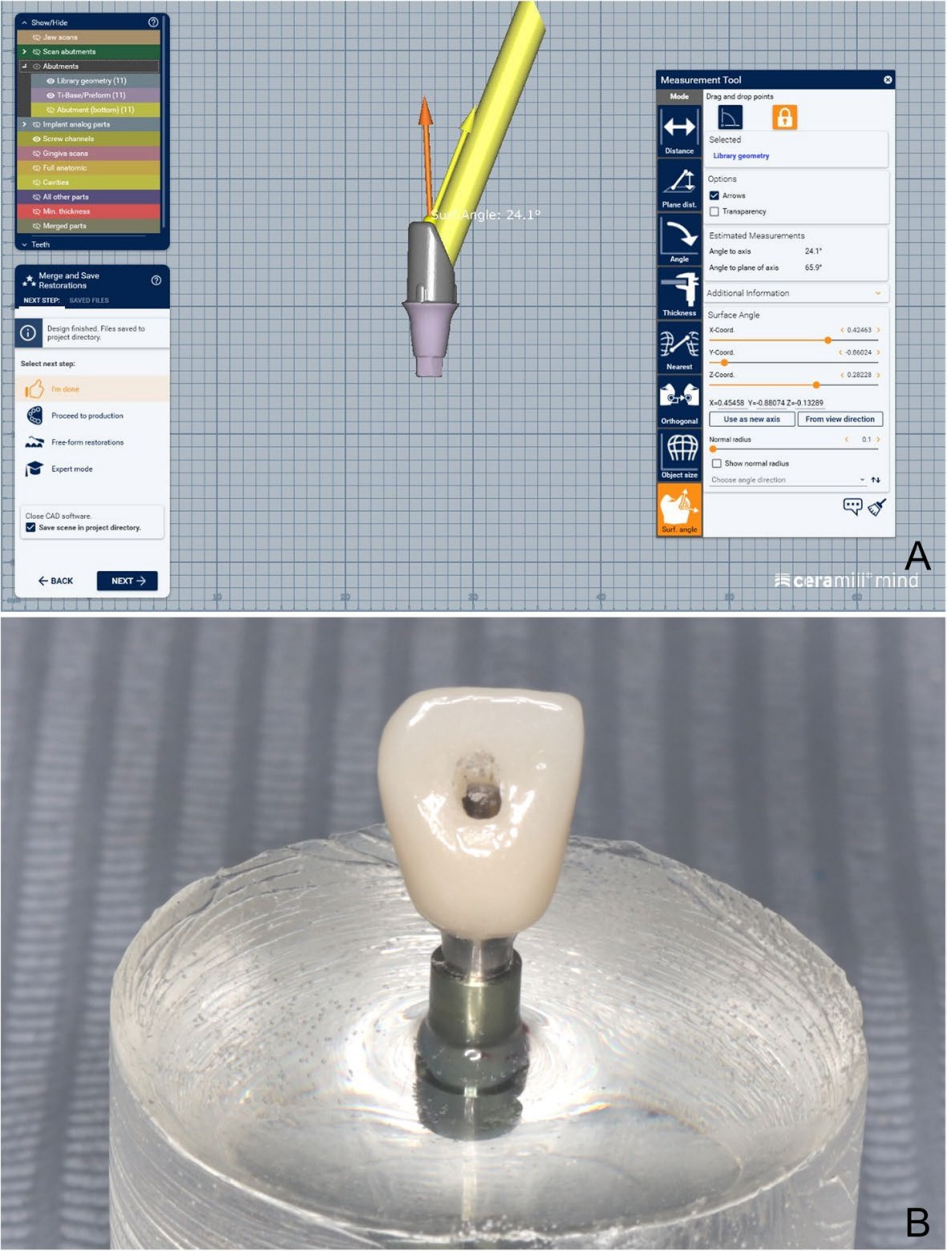
**A**, **B** Screw access hole of restoration for group ASC was located on palatal surface with angulation 24 degrees

For Group ASC, angled screw access channel Ti-base (GM titanium base AS, Neodent) of the same dimensions was selected from the library on (Ceramill, Amann Girrbach) CAD software. The screw access channel was situated on the palatal surface with angulation 24 degrees (Fig. 4 A, B).

The crowns of both groups were designed precisely for easier pull off the retention test.^5^ The STL files for the digitized crowns were exported to 5-axis milling machine (CORiTEC 150i dry, imes icore) for the milling process of zirconia crowns (Chengdu Besmile Biotechnology Co.,Ltd; yttrium oxide 4.5%).

**Fig. 5:**
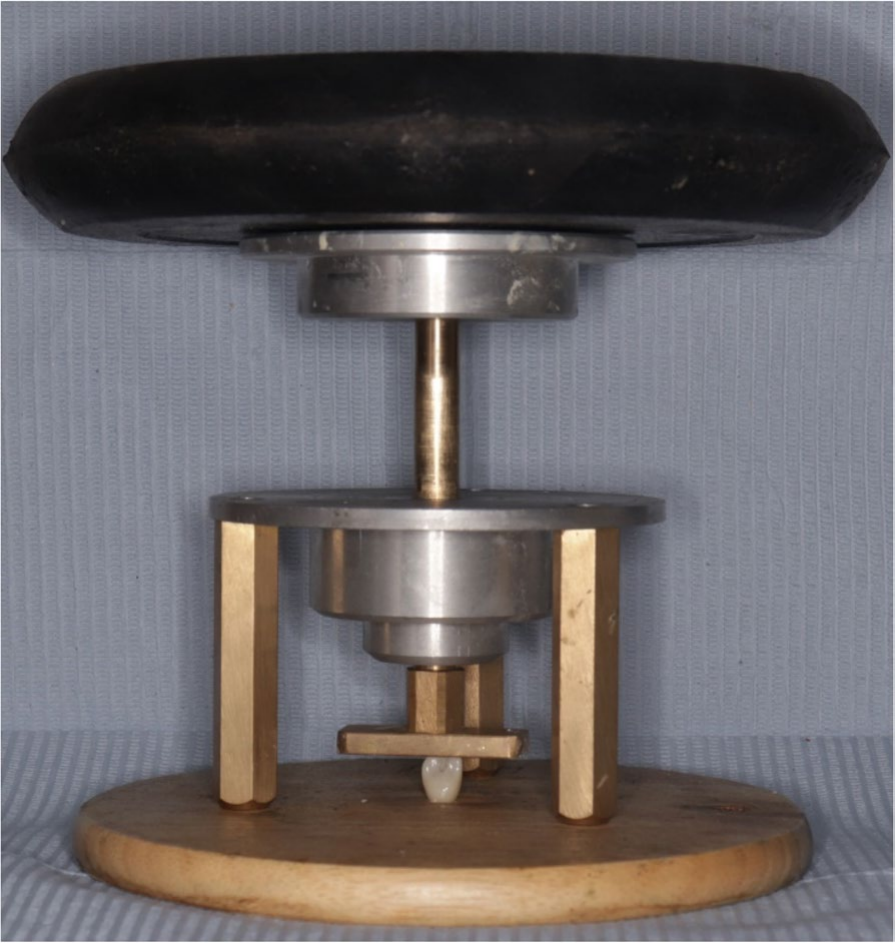
Load (25 N) was vertically applied for period of 10 min.*Caption:* Central vertical arm can be only raised for short distance to accommodate height of crown without epoxy resin block

Polytetrafluoroethylene tape was used to close the screw access hole. Aluminium oxide particles (120 µ) were used to sandblast the inner surface of the crown. The restoration was cleaned in an ultrasonic unit for approximately 1 min. The restoration was then thoroughly washed with water spray and dried with water/air followed by cleaning with Ivoclean. A single coat of primer was applied with a brush to the pre-treated surfaces. The primer was left for 180 s then was dissipated with a strong stream of air. Self-adhesive resin sealant (Multilink speed, Ivoclar Vivadent) was used as a bonding agent to bind zirconia crowns to their corresponding Ti-bases [16]. Spot curing for the margins for one second per quarter surface. After the elimination of excess cement, curing for the entire surface was carried out for 20 s. This procedure was conducted with firm finger pressure for 5 s and a 25 N load which was vertically applied for a period of 10 min [17] (Fig. 5). Subsequently, the excess cement was cleaned, and the one-piece restorations were screwed onto the implants with the torque recommended by the manufacturer (20 N). Retightening was done 10 min after the application of the first torque to compensate for preload loss because of settling effect of screw [18, 19]. Polytetrafluoroethylene tape and a composite filling material were used to close the screw access channels [16].

### Artificial aging

The samples were exposed to artificial aging through thermal cycling (5 °C to 55 °C, dwell time 60 s) according to ISO TR11405 (1994) and mechanical loading (250,000 cycles, 100 N, 1.67 Hz) in a mastication simulator, which is correspondent to relatively 1 year of masticatory cycles [20, 21]. An antagonist indenter was used in the shape of steatite ball of diameter 4 mm to load a force of 100 N at an angle on 2 mm from the incisal edge of the crowns.

### Pull-off retention test

**Fig. 6:**
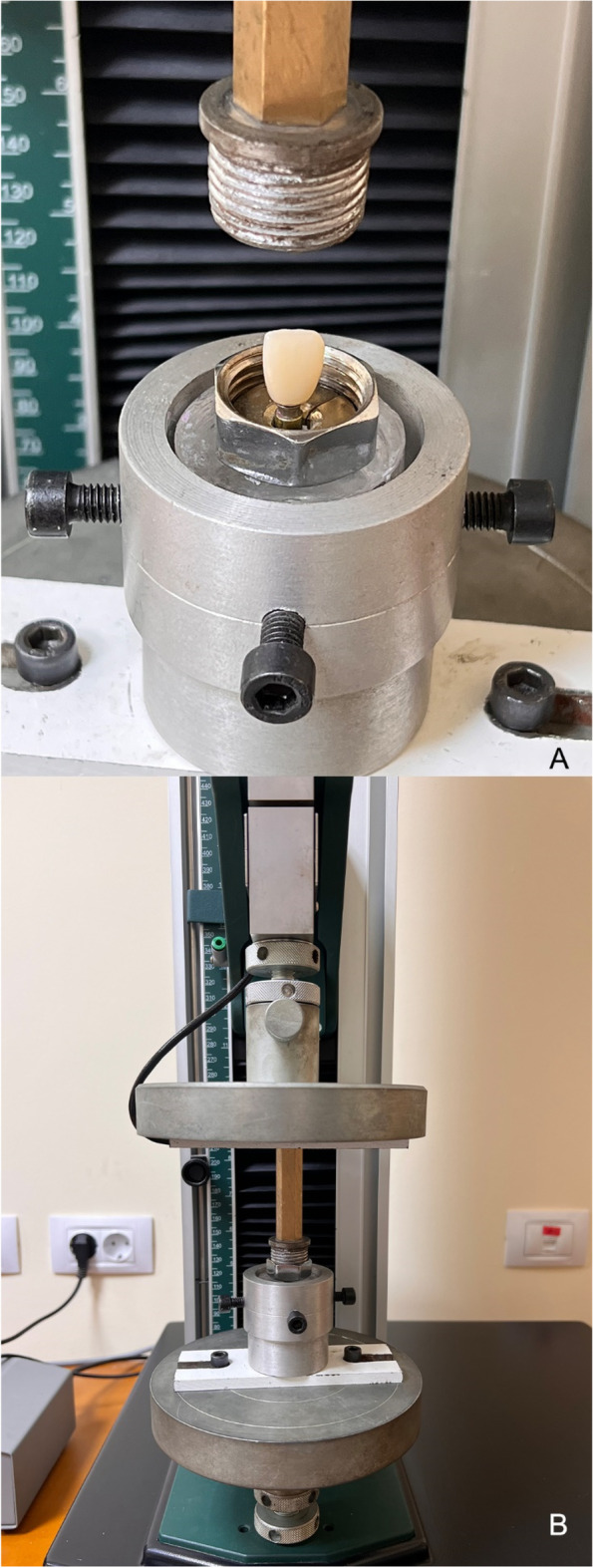
(**A**,** B**)** A**. Custom tensile device for axially seperating crowns from abutment surface. **B** Universal testing machine

Universal testing machine (5ST, Tinius Olsen) was used to implement the retention testing with a customized device for axially separating crowns from abutment surface at 1 mm/min crosshead speed [5, 17, 22]. The customized device was formed of two parts screwed to each other, having a split ring imbedded in between to support the neck of the crown (Fig. 6 A, B).

**Fig. 7:**
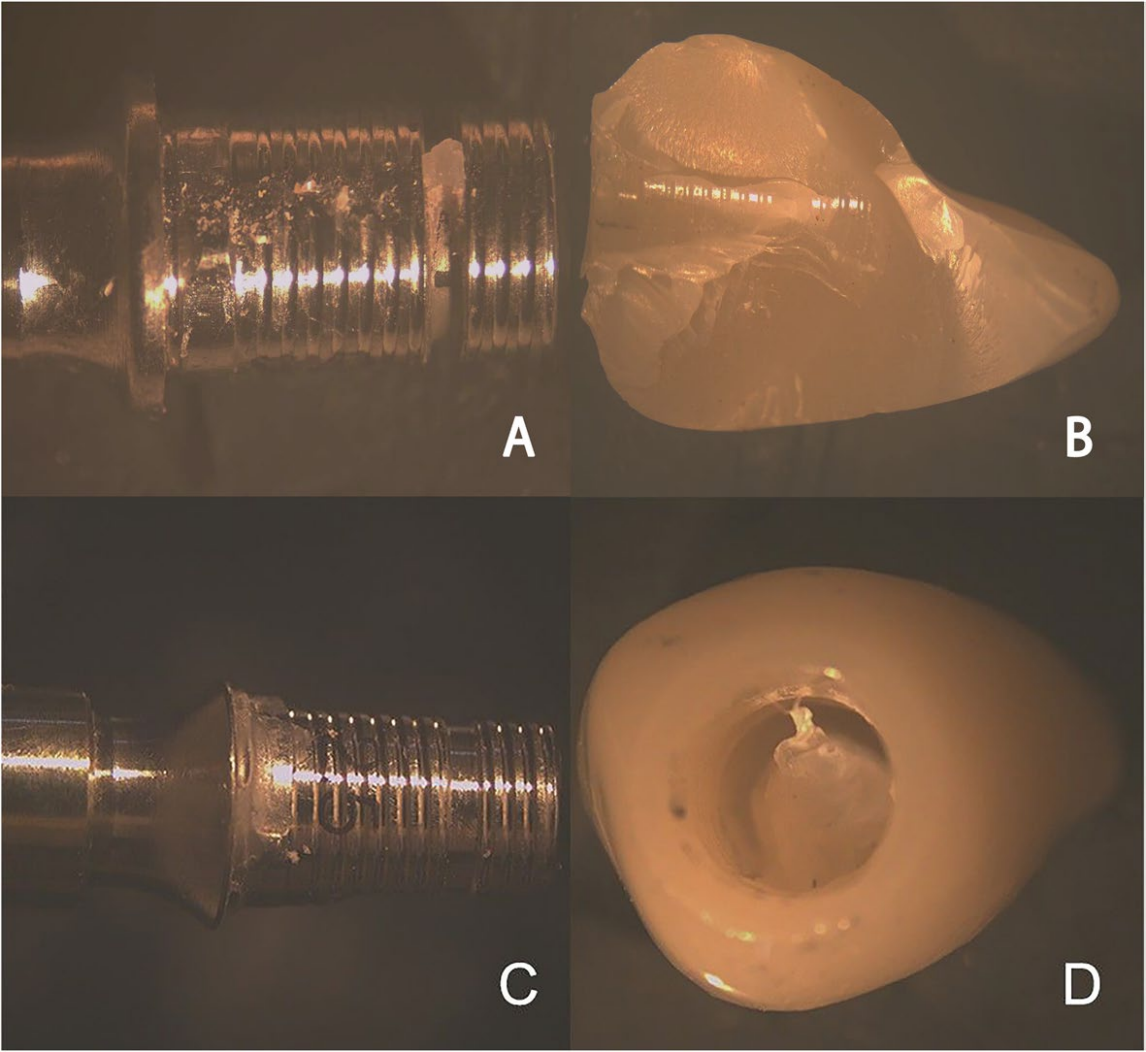
(**A**, **B**, **C**, **D**) Representative Stereomicroscope under × 20 magnification of remaining cement after retention test, (**A**,**B**) STA group, (**C**,**D**) ASC group

A software program (Horizon, version 12.2.4.0.) was used to register the records for the maximum pull-off force in newtons (N), for each sample. After the retention test, the abutment surfaces and the inner surfaces of the restorations were visually inspected with a Stereomicroscope (B016, Olympus, Japan; Software: Toup view, version 3.7) under × 20 magnification for remaining cement, and classification of failure modes (Fig. 7 A-D). Three categories for modes of failure were identified; Type 1, Adhesive failure when luting agent predominantly remained on the Ti-base surface (> 90%); Type 2, cohesive failure when luting agent remained on both Ti-base and crown surfaces; and Type 3, Adhesive failure when luting agent predominantly remained on the crown (> 90%) [23].

Statistical software (IBM SPSS version 28) was used for the statistical analysis to estimate the differences between the groups. Normality was checked by using Shapiro Wilk test and Q-Q plots. Values were normally distributed; therefore, groups were compared by using independent t-test. The tests were two-tailed, and the level of significance was set at *P* ≤ 0.05.

## Results

Mean value of retention forces in group STA (1731.57 N) was higher than that of group ASC (1032.29 N). Therefore, there is statistically significant difference between pull-off retention forces of straight and angled screw access channel abutments (*P* < 0.05) as illustrated in Table 1.

**Table 1:** Mean and standard deviation of retention forces between straight and angled screw access channel abutments

Groups	Straight screw access channel Ti-base (*n* = 7)	Angled screw access channel Ti-base (*n* = 7)
Mean (SD)	1731.57 (63.68) N	1032.29 (89.82) N
Mean difference (95% CI)	-699.29 (-608.62, -789.95)	
Effect size (d)	8.982	
Test (*P* value)	16.804 (< 0.0001*)	

^*^Statistically significant at *P* ≤ .05, CI: Confidence Interval

Visual inspection of all samples under × 20 magnification of stereomicroscope revealed Type 2 failure mode for all the samples in group STA along with Type 3 for group ASC.

## Discussion

The study revealed that angled screw channel abutment affected the pull-off retention forces on zirconia crown. In fact, the angled abutments caused reduction in the retention of zirconia restorations. Therefore, the null hypothesis was rejected.

The explanation to this result is the fact that, ASC abutment has larger screw access opening and fewer axial walls than the straight abutment as result of the greater angulation. Therefore, the surface area between the abutment and the crown is reduced causing further reduction in retention force.

The retention of prosthetic restorations is affected by many factors such as height of abutment, surface area, preparation geometry, surface roughness, and bonding agent [10, 24–26]. This comes in agreement with Tan et al. [9], who reported that extra surface area can be provided by increasing the number of axial walls. In addition, Muller L et al. [27], stated that abutment height is positively correlated to retention of permanent cements luting zirconia crowns to Ti bases and higher retention forces were reported for tall abutments compared to short abutments [8, 10, 11]

Bergamo ET et al. [28], stated that sandblasting the titanium surface can increase surface roughness, which provides an improved micromechanical interlocking by increasing surface area available for bonding, and such surface modifications enhanced the wettability of the bonding system.

In this study, two specimens of the straight abutments showed abutment screw fracture without crown-abutment debonding at 1350 N and 1590 N. One specimen of the straight abutment showed fracture of zirconia crown during retention test at 1810 N.

The retention forces, in this study, were of high values in comparison to other studies [17, 24, 28, 29] but were consistent with others [27, 30]. The reason may include the use of abutments with greater heights, titanium abutments with retentive areas and the type of self-adhesive resin sealant in addition to using primer which incorporate 10-methacryloxydecyl dihydrogen phosphate (MDP) [11, 17].

The forces needed to dislodge the crown were higher in the straight abutment than the angled abutment and the difference was found to be statistically significant. Further invivo studies are needed to identify the clinical significance of the recorded values.

Failure modes that occurred after the pull-off test showed mixed cement deposition on both of Ti-base and crown surfaces (Type 2) for the straight abutment group, indicating cohesive failure. While, in the angled screw access channel abutment, most cement remained on the intaglio of the crowns (Type 3), which identified an adhesive failure between the Ti-base surface and the luting agent. These observations assist the benefit of increasing means of retention to improve the adhesion of angled screw channel abutments to titanium surfaces.

In this study, the methodology was used to mimic the standard clinical situations. Thermocycling and mechanical loading were performed to replicate the oral environment [28]. Thermocycling is an identified form of aging, because hydrolytic degradation, repeated expansion/contraction, temperature changes, as well as other stresses within a sample have a significant impact on tensile strength [30]. Replicating the masticatory cycle is an essential factor in laboratory simulation [20]. Increased number of cycles in cyclic loading may also have an impact on monolithic zirconia crowns [31].

## Conclusions

The retention of zirconia crowns to abutments with a straight screw access channels is significantly higher than abutments with angled screw access channel. Angled screw access channel abutment causes reduction in the pull-off retention force of zirconia crown. It is recommended to perform further invivo studies to prove the invitro findings of this study.

## Data Availability

The datasets used and analyzed during the current RCT are available from the corresponding author upon reasonable request.

## Abbreviations

ASC: Angled screw access channel abutment
STA: Straight screw access channel abutment
Ti-bases: Titanium bases
N: Newtons
MDP: Methacryloxydecyl dihydrogen phosphate
